# Heterogeneous nonataxic phenotypes of spinocerebellar ataxia in a Taiwanese population

**DOI:** 10.1002/brb3.1414

**Published:** 2019-09-16

**Authors:** Szu‐Ju Chen, Ni‐Chung Lee, Yin‐Hsiu Chien, Wuh‐Liang Hwu, Chin‐Hsien Lin

**Affiliations:** ^1^ Department of Neurology National Taiwan University Hospital National Taiwan University College of Medicine Taipei Taiwan; ^2^ National Taiwan University Hospital Bei-Hu Branch Taipei Taiwan; ^3^ Department of Medical Genetics and Pediatrics National Taiwan University Hospital National Taiwan University College of Medicine Taipei Taiwan

**Keywords:** dystonia, ethnicity, parkinsonism, phenotypes, spinocerebellar ataxia

## Abstract

**Background:**

Spinocerebellar ataxia (SCA) presents with variable clinical presentations in addition to ataxia. The aim of this study was to reappraise the diverse nonataxic clinical characteristics of the five most common SCA subtypes in the Asian population.

**Methods:**

The clinical presentations of 90 patients with genetically confirmed SCA1, SCA2, SCA3, SCA6, or SCA17 were assessed retrospectively between November 2008 and September 2018 at a tertiary referral center in Taiwan.

**Results:**

Parkinsonism was the most common nonataxic phenotype (21.1%), with a greater prevalence than Caucasian and other Asian SCA carriers. Patients with parkinsonism feature had fewer CAG repeats in SCA2 (31.0 ± 4.5 vs. 36.9 ± 6.0, *p* = .03) and SCA3 (65.6 ± 7.9 vs. 70.0 ± 4.2, *p* = .02) compared to those with pure ataxia presentation. The average age of symptom onset was significantly higher in the parkinsonism group of SCA2 (51.5 ± 8.9 vs. 35.3 ± 12.6 years, *p* = .007) than those with pure ataxia. Focal or segmental dystonia was identified in 4.4% of SCA patients (*n* = 2 each SCA2 and SCA3). Nonmotor symptoms, including impaired cognition (6.1% of SCA2 and 8.3% of SCA3 patients) and depression (9.1% of SCA2 and 8.3% of SCA3 patients), were also common nonataxic features in our SCA patients.

**Conclusions:**

Parkinsonism, dystonia, and cognitive‐psychiatric symptoms are common features in patients with SCA mutations in our population. Our study identifies a different clinical spectrum of SCA1, SCA2, SCA3, SCA6, and SCA17 compared to Caucasians.

## Graphical abstract

We reappraise the diverse nonataxic clinical characteristics of the five most common spinocerebellar ataxia (SCA) subtypes in the Asian population. Parkinsonism, dystonia, and cognitive‐psychiatric symptoms are common features in patients with SCA mutations in our population. Our results would assist clinicians in identifying different subtypes of SCA patients.

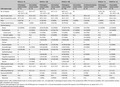

## 1 INTRODUCTION

Spinocerebellar ataxia (SCA) is a group of autosomal dominant hereditary cerebellar disorders with heterogeneous phenotypes (Sullivan, Yau, O'Connor, & Houlden, 2018). In addition to ataxia, nonataxic features, including movement disorders and dementia, are recognized in some SCA patients (Storey, 2016). Although these symptoms overlap in a wide range of SCA subtypes, the presence of specific nonataxic clinical presentations may be regarded as clinical clues to specific subtypes of SCA (Coarelli, Brice, & Durr, 2018; Rossi, Perez‐Lloret, Doldan, et al., 2014; Sullivan et al., 2018). For example, slow saccade is frequently identified in patients with SCA2, whereas seizure is more suggestive of SCA10 (Storey, 2016). Features of movement disorders are among the most common co‐existing nonataxic symptoms in SCA, especially SCA3 (Garcia Ruiz, Mayo, Hernandez, Cantarero, & Ayuso, 2002; Schols et al., 2000). These nonataxic clinical presentations have also been reported to predict clinical progression and prognosis (Kuo et al., 2017; Monte et al., 2017).

Although the number of pathological CAG repeats is the major determinant of phenotypic variation, the comorbid nonataxic phenotypes vary among diverse ethnic groups (Storey, 2016). For example, studies have shown that Asian patients with SCA2 may initially present as idiopathic Parkinson's disease (PD), but rarely in Western countries. In addition, SCA10 is frequently associated with epilepsy in Mexicans but manifests as pure cerebellar ataxia in Brazilians (Teive et al., 2011). Although these heterogeneous clinical manifestations are mainly attributed to the length of nucleotide repeats and presence of interrupted expansion affecting mRNA transcription and translation (McFarland et al., 2014; Rossi, Perez‐Lloret, Doldan, et al., 2014), other undefined genetic modifiers may affect the phenotype of SCA. Few studies have examined the full nonataxic clinical spectrum in patients with SCA, especially in Asians (Lee et al., 2011). Therefore, we aimed to reappraise the diverse nonataxic clinical characteristics of the five most common SCA subtypes in the East Asian population and to compare them with other ethnic groups.

## 2 METHODS

### 2.1 Patients

We retrospectively identified patients who had genetically confirmed mutations in SCA1, SCA2, SCA3, SCA6, or SCA17 at the Department of Medical Genetics in National Taiwan University Hospital between November 2008 and September 2018. Demographic data, clinical manifestations, clinical course, and the results of brain imaging studies and neurophysiological tests were obtained by reviewing the medical records of individual patients. Disease duration was defined as the interval between onset of initial neurological symptoms and the date that the genetic study was performed. Disease severity was measured using the modified Rankin Scale (mRS) when the genetic study was performed and at the last follow‐up in the hospital. The cognitive function and psychiatric status, including anxiety and depression, were retrieved from the medical records. All patients who were recorded to have cognitive decline received cognitive evaluation by the Mini‐Mental State Examination (MMSE) (Folstein, Folstein, & McHugh, 1975) with an MMSE score ≤25 being the cutoff for identifying a significant cognitive impairment, as well as impairments of instrumental activities of daily living (e.g., inability to manage finances and cope in social situations). Depression was evaluated by Beck Depression Inventory (Beck, Steer, Ball, & Ranieri, 1996), and anxiety was evaluated by Beck Anxiety Inventory (Beck, Epstein, Brown, & Steer, 1988). The Institutional Review Board of National Taiwan University Hospital approved this study and all the patients provided the informed consents.

### 2.2 Statistical analysis

Demographic data, clinical presentations, and functional status were compared between patients with different subtypes of SCA using the Kruskal–Wallis test for continuous variables and Fisher exact test for categorical factors. Using the independent *t* test, associations between the presence of parkinsonism and factors, including number of CAG repeats and age at symptom onset, were examined in patients diagnosed with SCA2 and SCA3. The characteristics of patients with and without parkinsonism were tested by an independent *t* test for continuous variables and chi‐squared test for categorical factors. We also compared the clinical features of SCA3 patients with and without simultaneous borderline repeat expansions in SCA2 using the Mann–Whitney test for continuous variables and Fisher exact test for categorical factors. Two‐tailed *p*‐values ≤.05 were considered significant. XLSTAT‐Biomed software was used for statistical analyses.

## 3 RESULTS

### 3.1 Demographics

A total of 187 patients (43.3% males) were genetically proven to carry mutations in SCA1, SCA2, SCA3, SCA6, or SCA17. SCA3 was the most common (*n* = 102, 54.5%), followed by SCA2 (*n* = 62, 33.2%), SCA1 (*n* = 11, 5.9%), SCA6 (*n* = 7, 3.7%), and SCA17 (*n* = 5, 2.7%). Notably, four patients who had pathological repeat expansion in SCA3 also simultaneously possessed an increased number of repeats in an intermediate range in SCA2 (Table 1). All the five patients with SCA17 have number of repeats between 41 and 43. The cutoff value for the pathologic CAG repeat number of SCA17 has not been clearly elucidated. Early reports proposed that SCA17 with a repeat number of 47 or more was suggested (Kim et al., 2009). However, the repeat number was then gradually lowered, and some later studies even suggested 41 repeats could be pathologic (Nanda, Jackson, Schwankhaus, & Metzer, 2007). In this study, the cutoff number for the pathological repeats of SCA17 is 49 and hence all the five patients presenting with SCA17 have repeat number in the intermediate range.

**Table 1:** Demographics, clinical characteristics, and number of CAG repeats for patients with SCA in the current study

CAG repeat range	SCA1 (*n* = 5)		SCA2 (*n* = 33)		SCA3 (*n* = 48)			SCA6 (*n* = 1)	SCA17 (*n* = 3)
	Pathological repeat number (*n* = 3)	Intermediate repeat number (*n* = 2)	Pathological repeat number (*n* = 27)	Intermediate repeat number (*n* = 6)	Pathological repeat number (*n* = 43)	Intermediate repeat number (*n* = 1)	Combined borderline repeats in SCA2^a^ (*n* = 4)	Pathological repeat number (*n* = 1)	Intermediate repeat number (*n* = 3)
No. of repeats	49.0 ± 1.7 (*N* ≤ 35, *P* ≥ 39)	36.5 ± 0.7	38.0 ± 4.1 (*N* ≤ 23, *P* ≥ 33)	25.7 ± 1.2	69.8 ± 4.1 (*N* ≤ 36, *P* ≥ 52)	45		21 (*N* ≤ 18, *P* ≥ 20)	41.7 ± 0.6 (*N* ≤ 40, *P* ≥ 49)
Age of onset, years	41.0 ± 2.6	41.0 ± 14.1	37.6 ± 13.5	49.3 ± 11.0	42.6 ± 17.2	53	39.0 ± 4.8	62	53.0 ± 8.5
Age of examination, years	45.7 ± 4.0	43.5 ± 10.6	42.9 ± 14.2	46.7 ± 19.8	46.2 ± 15.5	54	42.3 ± 6.8	64	46.0 ± 16.4
Male	2 (66.7%)	0	17 (62.9%)	3 (100%)	16 (37.2%)	1 (100%)	1 (33.3%)	1 (100%)	2 (66.7%)
Positive family history	3 (100%)	1 (50%)	23 (85.2%)	2 (33.3%)	34 (79.1%)	N.A.	2 (50%)	1 (100%)	1 (33.3%)
Clinical features									
Ataxia	3/3 (100%)	2/2 (100%)	23/24 (95.8%)	3/3 (100%)	37/38 (97.4%)	1/1 (100%)	3/4 (75%)	1/1 (100%)	2/2 (100%)
Parkinsonism	0	1/2 (50%)	4/24 (16.7%)	2/3 (66.7%)	5/38 (13.2%)	1/1 (100%)	4/4 (100%)	0	2/2 (100%)
Tremor pred.	N.A.	1/1 (100%)	1/4 (25%)	1/2 (50%)	1/5 (20%)	1/1 (100%)	3/4 (75%)	N.A.	N.A.
Akinetic‐rigidity	N.A.	N.A.	3/4 (75%)	1/2 (50%)	4/5 (80%)	N.A.	1/4 (25%)	N.A.	2/2 (100%)
Good levodopa response	N.A.	N.A.	3/4 (75%)	0	2/5 (40%)	0	4/4 (100%)	N.A.	0
Dystonia	0	0	1/24 (4.2%)	1/3 (33.3%)	1/38 (2.6%)	0	1/4 (25%)	0	0
Chorea	0	0	0	0	0	0	0	0	0
Polyneuropathy	0	0	8/24 (66.7%)	1/3 (33.3%)	14/38 (36.8%)	0	2/4 (50%)	0	1/2 (50%)
Pyramidal signs	1/3 (33.3%)	1/2 (50%)	5/24 (20.8%)	1/3 (33.3%)	6/38 (15.8%)	0	2/4 (50%)	0	0
Slow saccade	1/3 (33.3%)	0	14/24 (58.3%)	2/3 (66.7%)	11/38 (28.9%)	0	0	0	1/2 (50%)
Ophthalmoplegia	0	0	0	0	4/38 (10.5%)	0	0	0	0
Nystagmus	0	0	2/24 (8.3%)	0	19/38 (50%)	0	2/4 (50%)	0	1/2 (50%)
Cognitive impairment	0	0	1/24 (4.2%)	1/3 (33.3%)	4/38 (10.5%)	0	0	0	1/2 (50%)
Psychiatric symptoms	0	1/2 (50%)	2/24 (8.3%)	1/3 (33.3%)	3/38 (7.9%)	0	2/4 (50%)	0	1/2 (50%)
Seizure	0	1/2 (50%)	0	0	1/38 (2.6%)^b^	0	0	0	0
mRS at genetic study	1.7 ± 0.6	1.5 ± 0.7	1.8 ± 1.3	1.2 ± 1.6	1.7 ± 1.0	1.0	2.0 ± 1.4	2.0	2.0 ± 2.0
Follow‐up duration, months	18.1 ± 10.1	52.3 ± 63.1	31.3 ± 35.9	41.3 ± 37.5	29.9 ± 36.8	19.5	69.9 ± 49.6	16.9	17.3 ± 11.1
mRS at last follow‐up	2.0 ± 0.0	5.0 ± 1.4	2.4 ± 1.3	1.8 ± 2.2	2.3 ± 1.4	4.0	2.3 ± 1.3	2.0	2.7 ± 3.1
Received SSR/RRIV	0	0	2 (7.4%)	2 (33.3%)	8 (18.6%)	1	3 (75%)	0	1 (33.3%)
Normal	N.A.	N.A.	1 (50%)	N.A.	3 (37.5%)	1	3 (100%)	N.A.	N.A.
Abnormal	N.A.	N.A.	1 (50%)	2 (100%)	5 (62.5%)	N.A.	N.A.	N.A.	1 (100%)

Abbreviations: mRS, modified Rankin Scale; *N*, normal repeat number; N.A., not applicable; *P*, pathological repeat number; Pred. predominant; RRIV, R‐R interval variability; SCA, spinocerebellar atrophy; SSR, sympathetic skin response.

a Patients with pathological CAG repeats in the ATXN3 gene (no. of repeats 67.5 ± 2.6) and intermediate increased CAG repeats in the ATXN2 gene (no. of repeats 26.5 ± 1.7).

b The patient had post‐traumatic epilepsy.

Detail clinical information was available from 90 patients out of 187 patients with genetic confirmation, and we therefore analyzed the nonataxic clinical presentations in these 90 patients. We did not enroll asymptomatic carriers in this study. Among the 90 symptomatic carriers (47.8% males), the average age of symptom onset was 41.6 ± 14.9 years and age of examination 45.0 ± 14.4 years. Disease duration was estimated to be 8.4 ± 11.8 years. The mean initial mRS was 1.7 ± 1.2. After an average follow‐up of 32.3 ± 36.4 months, the mean mRS was 2.4 ± 1.5. Detailed demographic data, clinical presentations, genetic results, and functional status among different subtypes are given in Table 1.

### 3.2 Clinical presentation

Ataxia was the most common presentation in all patients with SCA (Table 1). Among symptomatic carriers, 83.3% initially manifested with ataxia; only three patients did not develop ataxia during the follow‐up period. Parkinsonism was the most common nonataxic movement disorder (21.1%; SCA1, *n* = 1; SCA2, *n* = 6; SCA3, *n* = 10; SCA17, *n* = 2), followed by focal or segmental dystonia (4.4%, *n* = 2 for both SCA2 and SCA3) manifested as blepharospasm, cervical torsion, striatal hand, and striatal foot. Slow saccade was the most common eye abnormality (32.2%) in our study. Nystagmus occurred in 26.7% of patients and presented significantly more often in SCA3 patients. Ophthalmoplegia was observed in 4.4% of patients and only noted in patients with SCA3 (Table 1).

Among the 19 patients presenting with parkinsonism, it was the initial neurological presentation in 10% of our studied patients (*n* = 9). Ataxia developed 14.9 ± 24.6 months later in seven patients. Most of the patients with parkinsonism were predominantly akinetic‐rigidity subtype (57.9%). Nearly half of the patients (*n* = 9, 47.4%) demonstrated clinical improvement after receiving levodopa. Notably, among these 19 patients, two patients were initially diagnosed with young‐onset PD and had a good response to levodopa treatment. Both patients underwent MRI examinations; one of them had mild cerebellar and brainstem atrophy, whereas the other had unremarkable findings. Patients with parkinsonism features had similar initial functional status but significantly worse mRS at last follow‐up compared to the group without parkinsonism (3.2 ± 1.4 vs. 2.6 ± 1.1, *p* = .04, Table 2). In the subgroup analysis based on individual type of SCA, patients with parkinsonism were associated with a fewer number of CAG repeats in SCA2 (31.0 ± 4.5 vs. 36.9 ± 6.0, *p* = .03) and SCA3 (65.6 ± 7.9 vs. 70.0 ± 4.2, *p* = .02). The average age of symptom onset was significantly older in the parkinsonism group of SCA2 patients (51.5 ± 8.9 vs. 35.3 ± 12.6 years old, *p* = .007) but not SCA3. Demographic data, clinical presentation, and functional status did not vary significantly among patients with parkinsonism carrying different SCA mutations (Table S1).

**Table 2:** Comparison of symptomatic SCA patients with and without parkinsonism

	With parkinsonism (*n* = 19)	Without parkinsonism (*n* = 59)	*p‐*Value
Age of symptom onset, years	47.1 ± 11.7	40.0 ± 15.6	.07
Gender, male	8 (42.1%)	27 (45.8%)	.78
mRS at genetic test	2.2 ± 1.2	1.9 ± 0.9	.33
Follow‐up period, months	46.3 ± 37.0	27.9 ± 34.8	.05
mRS at last follow‐up	3.2 ± 1.4	2.6 ± 1.1	.04
Brainstem atrophy on brain MRI	10/11 (90.9%)	19/31 (61.3%)	.07

Note

Data are presented as mean ± *SD* or *n* (%).

Abbreviations: mRS, modified Rankin Scale; SCA, spinocerebellar atrophy.

The nonmotor symptoms observed in our patients included cognitive impairment, psychiatric symptoms, and seizure. Cognitive impairment was noted in seven patients (SCA2, *n* = 2; SCA3, *n* = 4; and SCA17, *n* = 1). Among the various SCA subtypes, cognitive dysfunction was more common and severe with SCA17 (50% vs. 6.1% and 8.3% for SCA2 and SCA3, respectively; Table 1). We found no remarkable cognitive decline in patients with SCA1 and SCA6. Psychiatric symptoms were observed in 10 patients (SCA1, *n* = 1; SCA2, *n* = 3; SCA3, *n* = 5; and SCA17, *n* = 1), manifesting as depression (SCA2, *n* = 3; SCA3, *n* = 4) and visual and/or auditory hallucinations (SCA1, *n* = 1; SCA2, *n* = 1; SCA3, *n* = 2; and SCA17, *n* = 1). Seizures occurred in two of our patients, one of them was considered as having post‐traumatic epilepsy; the other patient had an intermediate increase in CAG repeats in SCA1 (Table 1). Autonomic function was measured by testing the sympathetic skin response and heart rate interval variation recorded in 17 patients (SCA2, *n* = 4; SCA3, *n* = 12; and SCA17, *n* = 1). Nine of the patients had abnormal results (75% of SCA2, 41.7% of SCA3, and 100% of SCA17 patients; Table 1), indicating autonomic system involvement (Table 1).

Notably, four patients who had pathological repeat expansion in SCA3 concomitantly had an intermediate number of repeats in SCA2. In these four patients, the proportions of parkinsonism (100% vs. 13.6%, *p* = .001) and psychiatric manifestations (50% vs. 6.8%, *p* = .049) were significantly higher compared to the other SCA3 patients without concomitant borderline increased repeats in SCA2 (Table 3). Most patients (75%) presented as tremor‐predominant subtype. Three of the four patients underwent examination by ^99m^Tc‐TRODAT single‐photon emission computed tomography and had asymmetric uptake in the basal ganglia. The parkinsonism features of all patients responded well to levodopa therapy (100%).

**Table 3:** Comparison of clinical characteristics and functional status between SCA3 patients with and without borderline repeat expansions in SCA2

	With borderline repeat expansions (*n* = 4)	Without borderline repeat expansions (*n* = 44)	*p‐*Value
Age of symptom onset, years	39.0 ± 4.8	42.9 ± 17.1	.610
Gender, male	1/4 (25%)	17/44 (38.6%)	>.99
Family history	2/4 (50%)	34/44 (77.2%)	.257
Sporadic onset	1/4 (25%)	3/44 (6.8%)	.302
Initial mRS	2.0 ± 1.4	1.7 ± 1.0	.865
Follow‐up, months	69.9 ± 49.6	29.6 ± 36.3	.185
Last mRS	2.3 ± 1.3	2.3 ± 1.4	.459
Clinical presentation			
Parkinsonism	4/4 (100%)	6/44 (13.6%)	.001
Ataxia	3/4 (75%)	38/44 (86.4%)	.480
Ophthalmoplegia	0	4/39 (10.3%)	>.99
Nystagmus	2/4 (50%)	19/39 (48.7%)	>.99
Slow saccade	0	11/39 (28.2%)	.558
Polyneuropathy	2/4 (50%)	14/44 (31.8%)	.592
Pyramidal sign	2/4 (50%)	6/27 (22.2%)	.268
Cognitive impairment	0	4/44 (9.1%)	>.99
Psychiatric symptoms	2/4 (50%)	3/44 (6.8%)	.049
Abnormal MRI study	0	19/24 (79.2%)	.019

Note

Data are presented as mean ± *SD* or *n*/total *n* (%).

Abbreviations: mRS, modified Rankin Scale; SCA, spinocerebellar atrophy.

## 4 DISCUSSION

We report the clinical spectrum of patients with different subtypes of SCA in Taiwan. Patients with a combined parkinsonism feature had fewer CAG repeats in SCA2 and SCA3 than patients presenting with ataxia only. The average age of neurological symptom onset among SCA2 patients was significantly older in the parkinsonism group than the group without parkinsonism. Nonmotor symptoms, including impaired cognition and depression, were also common features in our SCA patients.

The prevalence of SCA subtypes could be affected by multiple factors, including ethnicity, topography, and the method of genetic analysis (Filla et al., 2000; Kim & Cho, 2015; Radhakrishnan, Goyal, Srivastava, Shukla, & Behari, 2018; Venkatesh et al., 2018; van de Warrenburg et al., 2002). SCA1, SCA2, and SCA3 are the most common subtypes in Caucasians, whereas SCA2, SCA3, and SCA6 are more frequently observed in Asians (Filla et al., 2000; Kim & Cho, 2015; Soong, Lu, Choo, & Lee, 2001; van de Warrenburg et al., 2002). Consistent with one previous large series study in Taiwan (Soong et al., 2001), our study demonstrated that SCA3 was the most common subtype of SCA, accounting for 102 cases (54.5%), followed by SCA2 (62, 33.2%), SCA1 (11, 5.9%), SCA6 (7, 3.7%), and SCA17 (5, 2.7%). Notably, four patients who had a pathological number of repeats in SCA3 also had intermediately increased repeats in SCA2. In addition, this subgroup of SCA3 patients had an increased frequency of parkinsonism and depression compared to other SCA3 patients. However, the statistic power of this comparison is limited due to the small number of this subgroup of SCA3 patients with concomitant intermediate repeat number of SCA2. Our findings may suggest that, although a pathological number of CAG repeats in SCA3 was the major determinant of neurological dysfunction in these four patients, the addition of borderline repeats in SCA2 or other repeat expansion genes may play a role in the heterogeneity of phenotypes such as parkinsonism features, suggesting an underlying complex interaction among repeat expansion genes (de Castilhos et al., 2014; Tezenas du Montcel et al., 2014).

Parkinsonism is the most common nonataxic movement disorder phenotype in SCA and commonly associated with SCA2, SCA3, and SCA17 (van Gaalen, Giunti, & Warrenburg, 2011; Rossi, Perez‐Lloret, Cerquetti, & Merello, 2014). Asians and Africans demonstrate a higher prevalence of parkinsonism in SCA2 and SCA3 than Caucasians (van Gaalen et al., 2011; Park, Kim, & Jeon, 2015; Rossi, Perez‐Lloret, Cerquetti, et al., 2014). In agreement with previous studies (van Gaalen et al., 2011; Park et al., 2015; Rossi, Perez‐Lloret, Cerquetti, et al., 2014; Shan et al., 2001; Subramony et al., 2002), approximately one‐fifth of patients with SCA2 and SCA3 in this study presented with parkinsonism over the course of their disease. The prevalence was even greater than that reported in other Asian countries, such as Thailand and India (Boonkongchuen et al., 2014; Radhakrishnan et al., 2018). An intermediate number of CAG repeats were recognized as a risk factor for the clinical manifestation of parkinsonism in SCA2 and SCA3 (van Gaalen et al., 2011; Park et al., 2015; Rossi, Perez‐Lloret, Cerquetti, et al., 2014; Subramony et al., 2002). One postmortem brain pathology study showed significant neuronal loss and depigmentation in the substantia nigra rather than neuronal loss in the cerebellum of patients with borderline repeat expansions in SCA2 and SCA3 (Park et al., 2015), which could partly explain the occurrence of the parkinsonism phenotype and good response of parkinsonism features to levodopa therapy in our study. We also found that parkinsonism tended to manifest as akinetic‐rigidity type rather than tremor‐predominant type and one‐tenth of patients were clinically diagnosed with young‐onset parkinsonism without remarkable cerebellar ataxia. As intermediate to pathological repeat expansion in SCA2 and SCA3 accounts for 1% to 9% of patients with familial parkinsonism worldwide, especially in Asians (van Gaalen et al., 2011; Park et al., 2015), it is recommended that Asian patients with early‐onset parkinsonism and familial parkinsonism undergo genetic testing for SCA2 and SCA3. Furthermore, in addition to parkinsonism, several studies have revealed that the presence of an intermediate CAG repeat with an interruption by a CAA triplet in SCA2 was also associated with an increased risk of amyotrophic lateral sclerosis (ALS) (Wang, Gomes, Cashman, Little, & Krewski, 2014). Future research should focus on the mechanisms involved in the etiology of ALS among intermediate CAG repeat carriers of SCA2. It would be valuable to follow up families with SCA2 and SCA3 to observe the intrafamilial or interfamilial phenotype differences of carriers to have a better understanding of the mechanisms leading to phenotypic heterogeneity in the carriers of SCA2 and SCA3.

Cognitive dysfunction, predominantly involving executive ability, visuospatial performance, and memory, has been reported in SCA patients as a result of impairment in subcortical structures and connections between the cerebellum and cerebral cortex (Coarelli et al., 2018; Fancellu et al., 2013). Among the various SCA subtypes, cognitive dysfunction is more common and severe in SCA17 than other subtypes of SCA. Compared to previous studies showing a high prevalence of dementia in SCA2 patients, affecting 24% of patients in a German population and 42% in a Chinese population, only 6.1% of SCA2 patients in our study had cognitive decline (Schols et al., 1997; Tang et al., 2000). In addition, the prevalence of impaired cognition was 8.3% among SCA3 patients in our study population. Our findings are in concordance with previous studies demonstrating that cognitive impairment is common in SCA17 but rare in SCA6 (Kawai, Suenaga, Watanabe, & Sobue, 2009). Though cognitive dysfunction in SCA appears to represent frontal dysfunction, the mechanisms underlying cognitive dysfunction have not been clarified. Nevertheless, various lesions, including those in the cerebrocerebellar circuitry, cortico‐striatalthalamocortical circuitry, and frontal lobe, may influence cognitive function to various degrees for each subtype of SCA. In addition to cognitive decline, psychiatric symptoms, including depression, anxiety, and personality change, have been reported in SCA patients, especially SCA17 (Fancellu et al., 2013; Rossi, Perez‐Lloret, Doldan, et al., 2014). Postmortem brain examinations have revealed shrinkage and moderate loss of neurons with gliosis in the fronto‐temporal cortex, thalamus, caudate nucleus, and putamen, and moderate Purkinje cell loss in the cerebellum in patients with SCA17 (Bruni et al., 2004; Rolfs et al., 2003). Neuronal intranuclear inclusion staining with anti‐TATA box‐binding protein and antipolyglutamine was much more widely distributed throughout the brain gray matter in SCA17 than in other SCA subtypes (Rolfs et al., 2003). These neuropathological findings could partly explain the higher prevalence of cognitive‐psychiatric symptoms in SCA17 than in other types of SCA.

In our study, the prevalence of depression in SCA2 (9.1%) and SCA3 (8.3%) was similar to the Indian population (Stezin et al., 2018). A higher frequency of psychiatric symptoms has been reported in Western countries, where studies have reported that 19.1% of SCA2 and 13.4% to 66.7% of SCA3 patients have depressive symptoms (Cecchin et al., 2007; Schmitz‐Hubsch et al., 2011; Zawacki, Grace, Friedman, & Sudarsky, 2002). The discrepancies may partially result from the different methods applied in detecting depressive symptoms and the influence of ethnicity. Seizures, which are regarded as a hallmark of SCA10 in association with interrupted repeat mutations (McFarland et al., 2014), have also been reported in patients with SCA17 (De Michele et al., 2003). We did not identify a seizure phenotype in patients with SCA17. However, one patient carrying an intermediate repeat expansion in SCA1 presented with epilepsy before the onset of ataxic symptoms. This index patient with SCA1 had an autosomal dominant family history of presenting with neuropsychological symptoms of epilepsy, psychiatric disease, and early dementia. Though neuropsychological dysfunction is frequently related to SCA1, epilepsy is rarely observed (Kawai et al., 2009). Recently, a neuroimaging study in SCA1 patients revealed widespread atrophy involving the cerebral cortex and white matter in addition to the cerebellum and brainstem, which may partly explain the presentation of epilepsy in patients with SCA1 (Martins Junior et al., 2018).

Our study has several limitations. First, the relatively limited sample size with detailed clinical records and long‐term follow‐up may limit the understanding of the landscape of individual SCA subtypes in our population. Second, as this is a retrospective study, although we put forth a best effort in acquiring data, missing data are not avoidable. Third, the observations for SCA1, SCA6, and SCA17 require further validation due to the smaller sample sizes. Fourth, we only assessed cognitive function using the MMSE, a measurement of global cognitive function. Detailed neuropsychological tests evaluating individual cognitive domains are warranted for further cognitive assessments in SCA patients in the future. Finally, we did not analyze Scale for the Assessment and Rating of Ataxia (SARA) scores in all symptomatic patients as the main focus in this study is the nonataxic phenotypes. Future studies that combined both SARA scores and nonataxic phenotypes will be needed to know better about the possible different progression rates of ataxia and nonataxic features of SCA patients.

Our study provided nonataxic clinical characteristics of the five most common SCA subtypes in the East Asian population and may assist clinicians in identifying different subtypes of SCA patients.

## CONFLICTS OF INTERESTS

None.

## Data Availability

The data that support the findings of this study are available from the corresponding author upon reasonable request.
