# Supplementary material for: Heterogeneous nonataxic phenotypes of spinocerebellar ataxia in a Taiwanese population
Source: Brain Behav. 2019 Sep 16;9(10):e01414. doi: 10.1002/brb3.1414 (PMC6790309; doi:10.1002/brb3.1414)
Supplement: Supplementary file 1 [file BRB3-9-e01414-s001.docx]

**Supplemental Table 1.** Comparison of demographics, clinical presentation, and functional status among different subtypes of SCA in patients with parkinsonism.

|  | SCA1 (n=1) | SCA2 (n=6) | SCA3 (n=10) | SCA17 (n=2) | *P*-value |
| --- | --- | --- | --- | --- | --- |
| Age of onset, years | 51 | 51.5±8.9 | 42.9±13.3 | 53.0±8.5 | 0.539 |
| Gender, male | 0 | 2 (33.3%) | 5 (50%) | 1 (50%) | 0.889 |
| Akinetic-rigid | 0 | 4 (66.7%) | 5 (50%) | 2 (100%) | 0.351 |
| Tremor | 1 (100%) | 2 (33.3%) | 5 (50%) | 0 |  |
| Good levodopa response, n/total n (%) | N.A. | 3/5 (60%) | 6/8 (75%) | 0 | 0.545 |
| Initial mRS | 2 | 2.5 ± 1.2 | 1.8 ± 1.2 | 3.0 ± 1.4 | 0.344 |
| Follow-up, months | 96.9 | 30.4 ± 27.9 | 55.4 ± 40.6 | 23.5 ± 4.5 | 0.389 |
| Last mRS | 4 | 3.2 ± 1.5 | 3 ± 1.3 | 4.0 ± 2.8 | 0.835 |
| Abnormal MRI findings,  n/total n (%) | 1/1 (100%) | 3/5 (60%) | 3/8 (37.5%) | 2/2 (100%) | 0.062 |

Data are given as mean ± standard deviation or n (%) unless otherwise noted. Abbreviations: SCA, spinocerebellar atrophy; mRS, modified Rankin Scale; N.A., not available.
